# Supplementary material for: Simulating quantum light propagation through atomic ensembles using matrix product states
Source: Nat Commun. 2017 Nov 23;8:1743. doi: 10.1038/s41467-017-01416-4 (PMC5700945; doi:10.1038/s41467-017-01416-4)
Supplement: Supplementary file 1 — Supplementary Information [file 41467_2017_1416_MOESM1_ESM.pdf]

SUPPLEMENTARY NOTE 1 - ENTANGLEMENT ENTROPY IN MULTIPHOTON PROPAGATION

The use of matrix product states to study the propagation of photons in atomic ensembles is only possible if the entanglement and the related bond dimension of the system do not grow excessively with system size. Here we examine some toy models of EIT propagation to get a feeling for how entanglement and bond dimension behave depending on the number of photons in the system and the number of spins. As a basis of our toy models we assume that propagation occurs as a spin wave excitation from ground state $|\mathrm{g}\rangle$ to state $|\mathrm{s}\rangle$ and ignore any population in the excited state in the N-atom atomic ensemble.

The simplest possible situation occurs when we consider an ensemble of harmonic oscillators instead of spins. In this case a coherent pulse entering the system is converted to a coherent spin-wave excitation in the EIT medium with corresponding annihilation operator $\hat{a} = \sum_j \mathcal{E}_j b_j$ (b_j creates a spin excitation $|\mathrm{g}\rangle \rightarrow |\mathrm{s}\rangle$ at position j with amplitude $\mathcal{E}_j$ normalized such that $\sum_j |\mathcal{E}_j|^2 = 1$.) The coherent spin wave $|\alpha\rangle = e^{\alpha\hat{a}^\dagger - |\alpha|^2/2}|0\rangle$ is a product state of local coherent states $|\alpha\rangle_j = e^{\alpha\mathcal{E}_j^* b_j^\dagger - |\alpha\mathcal{E}_j|^2/2}|0\rangle_j$ and the maximum bond dimension for the representation of this state is 1.

The situation becomes more complicated when we treat instead spins, as more than one spin excitation cannot occur at the same site creating entanglement when more than one photon is present. In this case we may create spin-wave excitations in the medium by applying the operator $\hat{a}_\mathrm{s} = \sum_j \mathcal{E}_j \sigma^j_{\mathrm{sg}}$ multiple times to the ground state $|0\rangle = |\mathrm{g}\rangle^{\otimes N}$. For $\mathcal{E}_j$ normalized such that $\sum_j |\mathcal{E}_j|^2 = 1$, the first spin excitation $|1\rangle = \hat{a}^\dagger_\mathrm{s}|0\rangle$ is properly normalized, however higher number states require additional normalization factors $|n\rangle = (\hat{a}^\dagger_\mathrm{s})^n|0\rangle/\sqrt{n!\mathcal{N}_n}$, where $\mathcal{N}_n = 1 - \sum_j \sum_{m=2}^n |\mathcal{E}_j|^{2m}\binom{n}{m}$ accounts for the fact that no spin can be excited to state $|\mathrm{s}\rangle$ more than once.

We may then take $|\Psi\rangle = e^{-|\alpha|^2/2}\sum \alpha^n|n\rangle/\sqrt{n!}$ as an ansatz for the spin wave created by an input coherent pulse. In which case, a spin wave of form $|\alpha\rangle = e^{\alpha\hat{a}^\dagger_\mathrm{s} - |\alpha|^2/2}|0\rangle$, being the product of local states $|\alpha\rangle_j = (1 + \alpha\mathcal{E}_j^*\sigma^j_{\mathrm{sg}})|0\rangle_j$, no longer completely describes the system after coherent excitation. For example, at the two-photon level we would have to add a correction to this product state of the form $(1/\sqrt{\mathcal{N}_n} - 1)\alpha^2/2\sum_{j,l}\mathcal{E}_j\mathcal{E}_l\sigma^j_{\mathrm{sg}}\sigma^l_{\mathrm{sg}}|0\rangle$. This can be done by increasing the bond dimension of the matrix product representation from 1 to 4. Including the corrections at each higher n photon level requires an additional $n+1$ bonds

to describe the corrections to the product state as we will see in the following more general treatment.

We now continue to assume that the multiphoton wavefunctions are separable, i.e., that $\mathcal{E}(j,l) = \mathcal{E}(j)\mathcal{E}(l)$, but take the arbitrary form, i.e., $|\Psi\rangle = \mathcal{E}^{(0)}|0\rangle + \sum_j \mathcal{E}_j^{(1)}\sigma_{\rm sg}^j|0\rangle + \sum_{j>l}\mathcal{E}_j^{(2)}\mathcal{E}_l^{(2)}\sigma_{\rm sg}^j\sigma_{\rm sg}^l|0\rangle + \sum_{j>l>k}\mathcal{E}_j^{(3)}\mathcal{E}_l^{(3)}\mathcal{E}_k^{(3)}\sigma_{\rm sg}^j\sigma_{\rm sg}^l\sigma_{\rm sg}^k|0\rangle + \ldots$. The matrix product state representing $|\Psi\rangle$ can always be produced by applying the following matrix product operator with block diagonal form to the ground state

$$\mathcal{O}_j = \begin{pmatrix} \mathcal{I}^j & \mathcal{E}_j^{(1)}\sigma_{\rm sg}^j & & & & & & & & \\ 0 & \mathcal{I}^j & & & & & & & & \\ & & \mathcal{I}^j & \mathcal{E}_j^{(2)}\sigma_{\rm sg}^j & 0 & & & & & \\ & & 0 & \mathcal{I}^j & \mathcal{E}_j^{(2)}\sigma_{\rm sg}^j & & & & & \\ & & 0 & 0 & \mathcal{I}^j & & & & & \\ & & & & & \mathcal{I}^j & \mathcal{E}_j^{(3)}\sigma_{\rm sg}^j & 0 & 0 & \\ & & & & & 0 & \mathcal{I}^j & \mathcal{E}_j^{(3)}\sigma_{\rm sg}^j & 0 & \\ & & & & & 0 & 0 & \mathcal{I}^j & \mathcal{E}_j^{(3)}\sigma_{\rm sg}^j & \\ & & & & & 0 & 0 & 0 & \mathcal{I}^j & \\ & & & & & & & & & \ddots \end{pmatrix} \tag{1}$$

with

$$\mathcal{O}_1 = \Big(\mathcal{I}^1,\ \mathcal{E}_j^{(1)}\sigma_{\rm sg}^j + \mathcal{E}_j^{(0)}\mathcal{I}^1,\ \mathcal{I}^1,\ \mathcal{E}_j^{(2)}\sigma_{\rm sg}^j,\ 0,\ \mathcal{I}^1,\ \mathcal{E}_j^{(3)}\sigma_{\rm sg}^j,\ 0,\ 0,\ \ldots\Big) \tag{2}$$

$$\mathcal{O}_N = \Big(\mathcal{E}_N^{(1)}\sigma_{\rm sg}^N,\ \mathcal{I}^N,\ 0,\ \mathcal{E}_N^{(2)}\sigma_{\rm sg}^N,\ \mathcal{I}^N,\ 0,\ 0,\ \mathcal{E}_N^{(3)}\sigma_{\rm sg}^N,\ \mathcal{I}^N,\ \ldots\Big)^T. \tag{3}$$

Here each separable n photon Fock state is described by a submatrix with bond dimension $n+1$. This results from the $n+1$ ways the photons can be distributed to the left or right of the bond, where due to the separability each way corresponds to a unique vector in the Schmidt decomposition. The maximum bond dimension required to represent the state with up to $N_{\rm p}$ photons is then given by $\sum_{n=0}^{N_{\rm p}}(n+1) = (N_{\rm p}+3)N_{\rm p}/2$.

When the photon wavefunctions are no longer separable, as is generally the case, the Schmidt basis grows. If we consider dividing the system into equal left and right halves, then any pure state can be written $|\Psi\rangle = \sum_\lambda s_\lambda|\lambda\rangle_{\rm L}|\lambda\rangle_{\rm R}$. For an n photon Fock state there are once again $n+1$ ways to distribute the photons to the left or right side leading to $n+1$ different subspaces in which $|\lambda\rangle_{\rm L}$ and $|\lambda\rangle_{\rm R}$ can live. However, without separability these subspaces can no longer be described by a single Schmidt basis vector. Instead, the number of Schmidt basis vectors in each subspace is upper bounded by the total number of basis vectors $\binom{N/2}{n_{\rm R}}$, describing $n_{\rm R}$ photons distributed over the $N/2$ atoms on the right, and also

by the number of ways in which the photons can be arranged in the left side $\binom{N/2}{n-n_{\rm R}}$. The maximum number of Schmidt basis vectors required is then $\sum_{k=0}^{n} \min\left[\binom{N/2}{n-k}, \binom{N/2}{k}\right]$. Thus for example for 2 photons we would change from the maximum bond dimension being 3 for the separable case to $2 + N/2$ in general, likewise from 4 to $2 + N$ for three photons. For large N this scales with $N^{\text{floor}(n/2)}$.

The above scaling is for an arbitrary n-photon state and gives the maximum bond dimension required to exactly describe such a state. However in many cases keeping the entire Schmidt basis may not be required to accurately approximate the state. The question then becomes whether this scaling applies to physically relevant states arising in real experiments. For example, a pulse that has finite length less than the size of the atomic ensemble $N_{\rm pulse} < N$ would result in the bond dimension scaling with $N_{\rm pulse}$ rather than N. To go further we can check specific examples of photon wavefunctions that we expect to arise in an experiment. For example, in vacuum induced transparency the multi-photon parts of the input pulse get stretched at the medium's boundary to become heart shaped in the medium. We can estimate how such a distortion changes the bond dimension, by creating a physically motivated guess at the multiphoton wavefunction and then splitting the pulse in half and comparing the singular values with those of a separable pulse.

To describe the heart-shaped pulse in the medium we consider a system being driven by a coherent pulse $\mathcal{E}(t) = e^{-t^2/\sigma_t^2}$. The presence of a photon pair with coordinates $z_j > z_l$ then has amplitude depending on the field at two different retarded times so that $\mathcal{E}(z_j, z_l) = \mathcal{E}[t - z_l/(2v_{\rm g})]\mathcal{E}[t - z_l/(2v_{\rm g}) - (z_j - z_l)/v_{\rm g}]$, where the denominators of $2v_{\rm g}$ and $v_{\rm g}$ result from the different group velocities when two or one photons are inside the medium. Similarly, for three photons with positions $z_j > z_l > z_k$, we expect $\mathcal{E}(z_j, z_l, z_k) = \mathcal{E}[t - z_k/(3v_{\rm g})]\mathcal{E}[t - z_k/(3v_{\rm g}) - (z_l - z_k)/(2v_{\rm g})]\mathcal{E}[t - z_k/(3v_{\rm g}) - (z_l - z_k)/(2v_{\rm g}) - (z_j - z_l)/v_{\rm g}]$. We can then express these wavefunctions in terms of arbitrary bases for the left and right halves of the system, with the pulse at the center of the system, i.e., $|\Psi\rangle = \sum_{\lambda_{\rm R},\lambda_{\rm L}} \psi_{\lambda_{\rm R},\lambda_{\rm L}} |\lambda_{\rm R}\rangle|\lambda_{\rm L}\rangle$. From the singular value decomposition of the matrix $\psi_{\lambda_{\rm R},\lambda_{\rm L}}$ we then find the number of singular values s_λ required to represent the system and hence the entanglement entropy $S = -\sum_\lambda s_\lambda^2 \log(s_\lambda^2)$.

As a point of comparison we first do this calculation for a separable state of two photons $\mathcal{E}(z_j, z_l) = \mathcal{E}[t - z_j/(2v_{\rm g})]\mathcal{E}[t - z_l/(2v_{\rm g})]$ in an ensemble of $N = 100$ atoms, where we take $t = (N + 1)a/(4v_{\rm g})$ and $\sigma_t = 10a/v_{\rm g}$ so that the pulse is centered and fits well within the atom ensemble. Going through the above procedure gives three singular val-

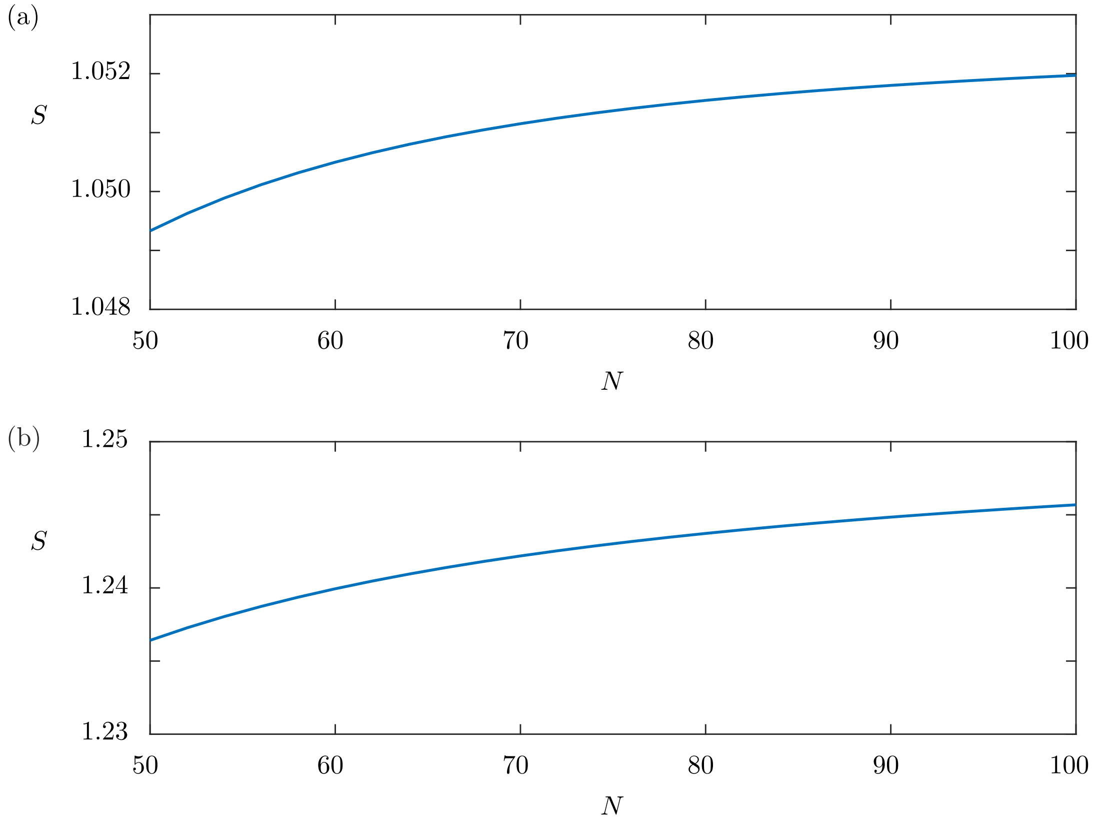

Supplementary Figure 1. **Growth of entanglement entropy in VIT.** Entanglement entropy for the (a) two-photon and (b) three-photon heart-shaped pulses described in the text as a function of the number of spins N in the spin chain.

ues as expected $s_\lambda^2 = 0.5145, 0.2427, 0.2427$ (the singular values are squared to correspond to probabilities), corresponding to $\sim 50\%$ of the state having one photon both in the left and right halves and $\sim 25\%$ having both photons in the left/right. The entanglement entropy across the central bond is then 1.03. For the two-photon heart shaped pulse given above, the number of singular values increases but by a small amount, with new singular values $s_\lambda^2 = 0.5000, 0.2623, 0.2355, 0.0022$ (the other values being at least a factor of 100 smaller) and entropy of entanglement of 1.05. For the three-photon separable case, where we now take $t = (N+1)a/(6v_{\rm g})$ to center the pulse, there are four singular values $s_\lambda^2 = 0.3822, 0.3822, 0.1178, 0.1178$ corresponding to having two photons on the left and one on the right and vice versa, and having all three photons on either the left or the right. The entanglement entropy in this case is 1.24. The heart shaped pulse on the other hand

has singular values $s_\lambda^2 = 0.4993, 0.2002, 0.1861, 0.1114, 0.0018, 0.0012$ (others a factor of 100 smaller) and entanglement entropy 1.25.

In Supplementary Figure 1 we show the dependence of the entanglement entropy on the number of atoms N for the two and three photon heart shaped pulses, where we have scaled the length of the pulse proportionally with the system size, $\sigma_t = aN/(10v_\mathrm{g})$. In this case we only observe a very small increase in the entanglement entropy with N with the value staying close to that expected for separable photon wavefunctions. We may then use the scaling $(N_\mathrm{p} + 3)N_\mathrm{p}/2$ found above in the separable case to estimate the bond dimension required to do VIT simulations if we want to keep up to N_p photons. For example, 10 photons would require a bond dimension of around 65. For general photon propagation problems, whether the bond dimension required is closer to the bound for the separable case or the non-separable case will be case dependent and determining if MPS treatments remain efficient for photons driven into highly correlated states needs further investigation.
